# Barriers to COVID-19 Vaccination and Perceptions Around Vaccination Uptake Strategies Among African Americans Living in the US South: Opportunities for Public Health Program Intervention

**DOI:** 10.1007/s40615-025-02433-6

**Published:** 2025-04-28

**Authors:** Biplav Babu Tiwari, Tatiana Woldman, Salma Sultana Resma, Jacob Matta, Heather Padilla, Janani Rajbhandari-Thapa

**Affiliations:** 1 Department of Health Policy and Management, College of Public Health, The University of Georgia, Athens, GA 30602 USA; 2 Department of Epidemiology and Biostatistics, College of Public Health, The University of Georgia, Athens, GA 30602 USA; 3 Department of Health Promotion and Behavior, College of Public Health, The University of Georgia, Athens, GA 30602 USA

**Keywords:** African American, COVID-19 vaccine hesitancy, Vaccine strategies, Rural health, Diffusion of innovation theory, Vaccine laggards

## Abstract

**Background:**

This study assesses the differences in vaccine hesitancy by vaccination status among African Americans (AAs) living in South Georgia and identifies preferred vaccine uptake strategies by the non-vaccinated AA.

**Methods:**

Survey data collected as a part of a COVID-19 Health Literacy program from adult (≥ 18 years) participants (*n* = 2058) in Albany, GA, was used (October 2022 to July 2023). We dichotomized COVID-19 vaccination status as “vaccinated” if reported having received at least one dose of vaccine, and “non-vaccinated” otherwise. Perception of vaccine barriers was assessed using 28 questions, and vaccine uptake strategies using 7 questions. All were assessed on a 5-point Likert scale, transformed to a dichotomous response, i.e., agree (merged strongly agree or agree responses) and disagree (merged strongly disagree, or disagree responses); neutral responses were dropped. Descriptive analysis and chi-square tests were used to identify the most prominent barriers to vaccination and the preferred uptake strategies among the non-vaccinated.

**Results:**

Nearly 1500 participants provided a non-neutral response to vaccine hesitancy questions, where the majority (90.7%) were vaccinated. Medical concerns and myth-related barriers were significantly associated with being vaccinated or non-vaccinated: for example, only 71.3% of non-vaccinated agreed that blood clots from the vaccine are of concern (a myth) compared to 40.5% of vaccinated (*p*-value < 0.001). Receiving additional information on the COVID-19 vaccine was selected as the most preferred strategy by the nonvaccinated.

**Conclusion:**

Medical concerns and myth-related barriers were the most common reasons for vaccine hesitancy, which could potentially be addressed by providing additional information on COVID-19 vaccination.

**Supplementary Information:**

The online version contains supplementary material available at 10.1007/s40615-025-02433-6.

## Introduction

The COVID-19 pandemic exacerbated health disparities; African Americans (AAs) were at higher risk of exposure to and impacts from COVID-19 due to their limited access to social, political, economic, and environmental resources [1, 2]. Cumulative age-adjusted data, as of May 2023, showed that patients identifying as AAs had two times higher rates of being hospitalized and had 1.6 times higher mortality rates than patients identifying as White [3]; this was especially the case in the younger age brackets with deaths in the 18–65 age range disproportionately affecting AA populations [4].

COVID-19 vaccination to the general public began on December 14, 2020; however, almost 50% of United States (US) counties had stagnant COVID-19 vaccination rates (less than 0.4% weekly growth rates) by May 2021, with the US south region having lower than national weekly growth rates [5]. Despite being disproportionately affected by the pandemic, communities with a majority AA saw low rates of increase in the proportion vaccinated for COVID-19 [6, 7]. Recent statistics show a continuation in COVID-19 vaccine hesitancy, as reported by the Centers for Disease Control and Prevention (CDC), as of May 11, 2024, only 19.9% of non-Hispanic AAs had received updated 2023–2024 COVID-19 vaccination compared to 22.2% of non-Hispanic Asians, and 25.6% of non-Hispanic Whites [8]. Therefore, there is a need in the public health literature to understand why communities with a higher proportion of AAs have been hesitant to receive COVID-19 vaccination. This is even more so important as new strains of the virus continue to affect our community over the 4 years since the first case of COVID-19 was confirmed in the US [9].

The diffusion of innovation (DOI) theory from the social sciences categorizes individuals or communities adopting an innovative intervention into five different categories based on time to adoption: innovators, early adopters, early majority, late majority, and the laggards [10]. Over 2 years after the release of the vaccine, the innovators, the early adopters, the early majority, and even the late majority have taken the COVID-19 vaccine, those remaining non-vaccinated may never be convinced of the vaccine’s safety and efficacy if no appropriate targeted interventions are implemented [11–14]. For example, even after the surge in delta-variant COVID-19 cases during the week of July 4, 2021, only one-third of US counties had increased weekly vaccination growth rates, and the stagnant counties had a very low vaccination growth rate (0.0033%) [5]. Recent studies on COVID-19 vaccination have not focused on the root causes of vaccine hesitation among communities that are not the innovators, the early adopters, or the majority according to the DOI theory (Appendix 1).

Evidence related to COVID-19 vaccine hesitancy highlights that mistrust of the medical establishment, clinical barriers such as uncertainty in vaccine safety, limited and inequitable access to healthcare resources, lower health literacy, and a history of racial discrimination are leading causes behind vaccine hesitancy in the AA community [15–18]. Concerns about vaccine safety, side effects, and vaccine efficacy are consistently found among the leading clinical causes of vaccine hesitancy [19, 20]. This evidence however is not generalizable across communities and is limited to studies with either lower samples of AAs, web-based surveys, studies without effectiveness assessment, or simply discussion papers only [15–18]. There is limited research examining barriers that potentially impact COVID-19 vaccination among AAs living in the US South.

Public health efforts to address vaccine hesitancy and thus increase COVID-19 vaccine uptake have been implemented using diverse approaches across various settings. Interventions include policy-level actions with incentives and disincentives, general and targeted communication campaigns and strategies, educational initiatives, financial incentives, and multidimensional approaches that combine several types of interventions [17, 21]. However, there is little literature comprehensively assessing the relative effectiveness of the existing vaccination promotion efforts. This is especially true when we look at efforts that specifically target the AA community. Most studies involving this population have a small sample size and address vaccine intention rather than vaccine behavior. Further, most of the studies come from the early days of vaccine development and deployment and therefore may no longer address the issues faced by communities and populations that have not been vaccinated yet 4 years after the global COVID-19 pandemic (Appendix 1). The pandemic took about 1.1 million lives in the US, as of June 8, 2024 [22], and recurring waves of variants (omicron, delta, and so on) [23] have continued to take more lives.

In this study, we investigate vaccine hesitancy among AAs who are yet non-vaccinated. We compare vaccine hesitancy perception and the preferred vaccine uptake strategies between the non-vaccinated and the vaccinated to inform ongoing efforts to promote vaccination. We first study differences in the intensity of hesitation among AAs who have received at least one dose of the COVID-19 vaccine with those who are non-vaccinated. We study vaccine hesitation related to clinical, access, trust, information, and religious barriers and hypothesize that the clinical barriers are the most prominent barriers to vaccination, based on a priori review of the literature [19, 20]. We further hypothesize that the vaccination uptake strategies to address these barriers will also differ between the two groups. The study focuses on a population sub-group who have remained non-vaccinated to identify the most preferred vaccine uptake strategies and differences by willingness to get vaccinated. The study provides us an opportunity to identify what vaccine uptake strategies have worked among AAs who have been vaccinated and, most importantly, identify potential vaccine uptake strategies perceived as most effective to influence AAs who are yet non-vaccinated to consider being vaccinated.

## Methods

### Study Design and Participants

The study used a cross-sectional design and collected survey data as a part of an evaluation effort of a community-based program to address COVID-19 vaccine hesitancy in the US South. The program was implemented in a socially vulnerable city in the US South Georgia (Albany, GA) with a majority AA population (75.6%) [24]. The city was one of the hardest-hit areas during the first wave of the COVID-19 pandemic in early 2020 [25]. However, despite 4 years since the first wave of COVID-19 and 3 years past the availability of COVID-19 vaccines, only 59% of the population in the county had received at least one dose of COVID-19 vaccine, compared to the state average of 68.3% as of January 24, 2024 [26, 27]. Further, as of January 24, 2024, only 51.6% of AAs in the program site had received at least one dose of COVID-19 vaccination. This prompted increased attention to increase access to health services, promote health literacy specific to COVID-19, and decrease vaccine hesitancy in the city.

The project was funded by the Office of Minority Health, Health, and Human Services (Grant #1 CPIMP211229 - 01–00) and was implemented in partnership between the city and academic institutions serving the city. The project was determined not involving human subjects by the Institutional Review Board at the [University Name] (IRB ID PROJECT00006561). The program took place between October 2022 and December 2023 in partnership with community organizations like churches, local healthcare providers, and youth organizations to deliver information about COVID-19 and COVID-19 vaccinations to residents of the city. Program events included discussion groups, workshops, health screenings, vaccine clinics, educational events, and fitness classes.

The data for this study was collected between October 2022 and July 2023, resulting in a total of 2058 survey participants. Further, per the priorities of the community-based programs, almost 95% of the survey sample identified as AAs. This study included responses from participants identifying as AAs to identify the most prominent barriers to vaccination and potential vaccine uptake strategies among this population sub-group. The final sample included in this study was 1471. Appendix 2 outlines the study’s sample selection procedure. Appendix 3 shows the distribution of study participants by their residence zip code (vaccinated and non-vaccinated are shown using different colors). The cluster of participants observed was in Albany, GA, i.e., the study site (magnified for legibility).

### Study Measures

#### COVID-19 Vaccination Status

The participants’ COVID-19 vaccination status was determined based on the responses to the following two questions in the survey: “Did you also receive a COVID-19 vaccine today?” and “Were you vaccinated in the past?” with possible responses “yes” and “no.” If participants had answered “yes” to either of the questions, i.e., administered at least one dose either in the past or during the event, they were categorized as “vaccinated,” and if they had answered “no” to both questions, i.e., never administered a COVID-19 vaccine, they were categorized as “non-vaccinated.”

#### Willingness to Vaccinate

We determined the willingness to vaccinate in the future among the “non-vaccinated” based on the response to “Do you plan to get vaccinated in the near future?” with possible responses “yes,” “no,” “not sure,” and “do not know.” If participants had answered “yes” to the question, they were categorized as “willing” and as “non-willing” if they answered other than “yes.” Similarly, among the vaccinated, we identified those who received their first dose of COVID-19 vaccination during the health literacy program based on the response to “Was the COVID-19 vaccine dose you received today your 1 st dose?” with possible responses “yes” and “no.” Those who answered “yes” were identified as receiving their first dose of COVID-19 vaccination during the health literacy program and were categorized as “willing.” We categorized those who received their first vaccination at the program site as willing because their decision to get vaccinated during the program event demonstrates a willingness to get vaccinated.

#### Vaccine Barriers and Vaccine Uptake Strategies

Our main predictors of interest were the perception of vaccine barriers and vaccine uptake strategies. There were 28 questions on vaccine barriers, categorized into six domains (number of questions under each domain provided in parenthesis), i.e., clinical (6), myths (4), access (4), information (4), trust (5), and religious (5) (Appendix 4). The vaccine uptake strategies were asked in 7 questions (Appendix 5). In the study, each question was treated as an individual barrier to vaccination and was assessed on a 5-point Likert scale, i.e., strongly disagree, disagree, neutral, agree, and strongly agree. These measures were adopted from existing literature on vaccine barriers [28–32] and uptake strategies [33, 34] and have been commonly used in studying COVID-19 vaccine hesitancy such as by Coman et al. [35]. The questions on vaccine barriers were administered in a negative direction; thus, “agree” and “strongly agree” responses would indicate perceiving those barriers as a reason behind being non-vaccinated and vice-versa. Whereas the questions on vaccine uptake strategies were administered in a positive direction, thus, “agree” and “strongly agree” responses would indicate perceiving those strategies as good actions for promoting vaccination. Finally, we dichotomized these Likert responses into two categories: “agreement” if answered strongly agree or agree and “disagreement” if answered strongly disagree or disagree, and we excluded neutral responses. The decision to exclude the neutral responses was guided by the study’s aim to identify the vaccine barriers and uptake strategies that the study participants truly agree/disagree with. By definition, the neutral responses in a Likert scale represent views where a participant cannot decide on either side of the response continuum and studies have found that respondents validly do so [36].

### Analysis

We conducted a descriptive summary of the study participants’ sociodemographic characteristics, i.e., gender, age, education, income, presence of comorbidities, and the presence of children and elderly in the household by vaccination status. It was followed by a non-parametric test using chi-square tests to study the differences between the groups.

We then performed a bivariate analysis of vaccination status with vaccine barriers and uptake strategies and used chi-square tests to conduct tests of significant differences regarding perceptions of vaccine barriers and uptake strategies between the two groups to test the observed differences between the two groups against the assumption of no difference. The analysis was done separately for each question with complete cases for that question. To identify the most prominent barrier, we compared the proportion of participants who agreed and disagreed with each vaccine barrier question within the vaccinated and non-vaccinated sub-sample. For example, one of the barriers was “Blood clots from the COVID-19 vaccine are of concern.” and if we observe a higher percentage of agreement (strongly agree or agree) (more than 50%) among non-vaccinated, then it would mean that the majority of the non-vaccinated agreed with the barrier. Next, we ordered the vaccine barriers by agreement proportion in descending order within each domain of vaccine barrier and observed if the majority vaccinated and majority non-vaccinated either both agreed to the barrier or both disagreed with the barrier, or one agreed and another disagreed with the barrier.

We followed a similar approach to identify the preferred vaccine uptake strategies, where we first compared the proportion of participants with agreement or disagreement to an uptake strategy by vaccination status and then compared it across each other. We also analyzed vaccine uptake strategies by demographic characteristics for the non-vaccinated group to identify if a specific strategy would emerge as the most preferred vaccination uptake strategy among specific sociodemographic groups. Finally, we also compared the vaccination strategies by willingness to vaccinate to identify the preferred strategies among those willing and non-willing to get vaccinated in the future.

All the bivariate comparisons were tested using chi-square tests at a 0.05 level of significance. R version 4.1.0 was used to conduct data analysis with the RStudio integrated development environment.

## Results

The majority of the participants were females (65.7%), had a high school level of education (75.2%), had no comorbidities (69.3%), and had no children (70.8%) or older adults (81.0%) in their household. Nearly 91% of the program participants had received at least one dose of vaccination. The majority of non-vaccinated participants were in the age group 18–34 whereas the majority vaccinated were in the age group 35 and over. Higher proportions of non-vaccinated individuals reported low income (47.4% with income less than $25,000 and 40.1% with income in the range $25,0000–$50,000) compared to vaccinated individuals. A higher proportion of participants who were vaccinated did not have children in the household (73.2% vs. 26.7%) compared to non-vaccinated (53.3% vs. 46.7%). The majority of the participants in both the vaccinated and non-vaccinated groups did not have the elderly in the household. Within both vaccinated and non-vaccinated groups, there was a higher proportion of participants with no chronic condition. Age, education, income, having children or elderly in the household, and having chronic conditions were significantly associated with being vaccinated (Table 1).

**Table 1:** Demographics by vaccination status, expressed as*N* (%)

	Vaccinated 1334 (90.7)	Non-vaccinated 137 (9.3)	Total (*N* = 1471)	*p*-value
Gender				
Male	463 [34.7]	41 [29.9]	504 [34.3]	0.345
Female	871 [65.3]	96 [70.1]	967 [65.7]	
Age group				
18–24	147 [12.8]	38 [34.9]	185 [14.7]	< 0.001
25–34	162 [14.1]	32 [29.4]	194 [15.5]	
35–64	536 [46.8]	26 [23.9]	562 [44.8]	
65 +	301 [26.3]	13 [11.9]	314 [25.0]	
Education				
Less than high school	124 [12.5]	21 [17.2]	145 [13.0]	0.018
High school	743 [75.0]	94 [77.0]	837 [75.2]	
Bachelor’s degree or higher	124 [12.5]	7 [5.7]	131 [11.8]	
Income				
Less than 25,000$	525 [39.4]	65 [47.4]	590 [40.1]	0.011
25,000$–49,900$	480 [36.0]	55 [40.1]	535 [36.4]	
50,000$–99,900$	255 [19.1]	14 [10.2]	269 [18.3]	
100,000$ or higher	74 [5.5]	3 [2.2]	77 [5.2]	
Has children				
Yes	356 [26.7]	73 [53.3]	429 [29.2]	< 0.001
No	977 [73.2]	64 [46.7]	1041 [70.8]	
Has elders				
Yes	262 [19.8]	15 [10.9]	277 [19.0]	0.007
No	1061 [80.2]	122 [89.1]	1183 [81.0]	
Has chronic condition				
No condition	887 [66.5]	116 [84.7]	1003 [69.3]	
At least 1 condition	447 [38.7]	21 [15.3]	468 [30.7]	< 0.001

### Vaccine Barriers

The perception of vaccine barriers among the vaccinated and non-vaccinated participants was significantly different within the clinical and myths domain. We observed an opposite direction for all barriers in the clinical domain and two barriers in the myth’s domain; the majority of the non-vaccinated group agreed with the barriers, whereas less than 50% of the vaccinated group agreed with the barriers (Table 2). For all questions related to access, information, trust, and religious barriers, both vaccinated and non-vaccinated groups responded in the same direction; less than 50% of participants agreed with statements about access, information, trust, and religious barriers.

**Table 2:** Perceptions of vaccine barriers by vaccination status (expressed as*N* (%) for agreeing or strongly agreeing to each type of barrier)

Vaccine barriers	Vaccination status		
	Vaccinated (*n* = 1334)	Non-vaccinated (*n* = 137)	*p*-value
Clinical barrier			
Blood clots from the COVID-19 vaccine are of concern	402 [40.5]	67 [71.3]	< 0.001
Dying from the COVID-19 vaccine is of concern	436 [41.2]	67 [69.8]	< 0.001
Getting sick from multiple doses of COVID-19 vaccine is of concern	396 [38.9]	66 [69.5]	< 0.001
Heart disease from the COVID-19 vaccine is of concern	389 [38.8]	60 [65.9]	< 0.001
Pain from/related to the COVID-19 vaccine is of concern	369 [38.7]	55 [65.5]	< 0.001
High blood pressure from the COVID-19 vaccine is of concern	362 [36.6]	51 [60.7]	< 0.001
Myths			
The COVID-19 vaccine leads to miscarriages and is not appropriate for pregnant women	280 [30.0]	48 [59.3]	< 0.001
COVID-19 vaccine components remain in the human body for a long period	362 [40.5]	47 [58.8]	0.002
COVID-19 vaccine implants a “computer chip” in the body	201 [19.6]	40 [45.5]	< 0.001
COVID-19 vaccine is NOT an effective way to protect against COVID-19	173 [16.3]	30 [39.5]	< 0.001
Access barrier			
It is inconvenient to get the COVID-19 vaccine	211 [19.2]	21 [24.1]	0.333
The clinics/venues that provide the COVID-19 vaccine are too far away	88 [9.0]	12 [12.8]	0.309
There is a shortage of the COVID-19 vaccine	117 [11.0]	11 [12.2]	0.859
I don’t know how to get ahold of the COVID-19 vaccine	121 [11.0]	10 [10.3]	0.974
Information barrier			
I don’t have enough information to decide whether to take COVID-19 vaccine or no	93 [8.9]	16 [17.6]	0.011
I don’t know where I can get accurate information about the COVID-19 vaccines	80 [7.7]	15 [16.0]	0.010
I am confused by the information about the COVID-19 vaccines	80 [7.8]	13 [14.4]	0.046
I don’t know where I can get trustworthy information about the COVID-19 vaccines	71 [6.9]	13 [13.8]	0.025
Trust barrier			
I don’t trust vaccines in general	89 [9.1]	32 [40.0]	< 0.001
I don’t trust the government agencies that approved the COVID-19 vaccines	97 [10.2]	30 [38.0]	< 0.001
I don’t trust media that recommend the COVID-19 vaccines	106 [11.2]	30 [37.5]	< 0.001
I don’t trust the pharmaceutical companies that manufacture the COVID-19 vaccines	92 [9.7]	29 [36.7]	< 0.001
I don’t trust the scientists/professionals who recommend the COVID-19 vaccines	81 [8.3]	23 [28.4]	< 0.001
Religious barrier			
As long as I am faithful to my God and/or my religion, I am protected from COVID-19, therefore I do not need the COVID-19 vaccine	131 [14.1]	41 [48.2]	< 0.001
It is better to use natural preventive methods (e.g., essential oils, other natural drinks, tonics, etc.) to prevent getting infected with COVID-19 than getting the vaccine	109 [11.6]	25 [33.8]	< 0.001
It is better to use spiritual/holy preventive measures (e.g., holy water, holy oil, cross, holy amulets, etc.) to prevent getting sick from COVID-19 than to get the COVID-19 vaccine	91 [9.7]	21 [26.9]	< 0.001
It is better to get sick of COVID-19 and for your body to fight it off, building natural immunity than getting the vaccine	110 [11.0]	19 [25.0]	< 0.001
The COVID-19 vaccine ingredients are banned by my religion, therefore, I cannot get the COVID-19 vaccine	67 [7.1]	15 [20.0]	< 0.001

### Vaccine Uptake Strategies

The perception of vaccine uptake strategies among the vaccinated and non-vaccinated participants was similar, where additional information on COVID-19 vaccination and recommendations from healthcare providers were identified as the top two strategies for vaccine uptake (Table 3).

**Table 3:** Differences in agreement to different vaccine uptake strategies by vaccination status, expressed as*N* (%)

Vaccine uptake strategies	Vaccination status		
	Vaccinated (*n* = 1334)	Non-vaccinated (*n* = 137)	*p*-value
I need to learn more about COVID-19 vaccination, its side effects, benefits, and future implications	848 [84.7]	60 [69.8]	< 0.0001
I am willing to get vaccinated if my healthcare provider recommends me to get the COVID-19 vaccine	957 [88.4]	61 [66.3]	< 0.0001
The CDC/local health department recommended us to get the COVID-19 vaccine	971 [89.2]	58 [65.9]	< 0.0001
I am willing to get vaccinated if my family members recommended me to get the COVID-19 vaccine	894 [86.9]	57 [62.6]	< 0.0001
I am willing to get vaccinated if my nurse recommended me to get the COVID-19 vaccine	853 [83.5]	60 [62.5]	< 0.0001
I am willing to get vaccinated if my colleagues/co-workers recommended me to get the COVID-19 vaccine	759 [79.4]	53 [58.9]	< 0.0001
I am willing to get vaccinated if my spiritual advisor (such as pastor/priest/rabbi/imam) recommended me to get the COVID-19 vaccine	818 [82.8]	48 [54.5]	< 0.0001

In a further investigation of the vaccine uptake strategies within the non-vaccinated group by their sociodemographic characteristics (Appendix Table 6), we found a higher proportion of participants expressed preference for the strategy “I need to learn more about COVID-19 vaccination, its side effects, benefits, and future implications” regardless of gender, age, or education. Another preferred strategy was “the CDC/local health department recommended us to get the COVID-19 vaccine.” However, strategies such as “I am willing to get vaccinated if my spiritual advisor (such as pastor/priest/rabbi/imam) recommended me to get the COVID-19 vaccine” and “I am willing to get vaccinated if my colleagues/co-workers recommended me to get the COVID-19 vaccine” were consistently least preferred.

### Willingness to Vaccinate

Among those willing to get vaccinated in the future, the most preferred strategy was “I need to learn more about COVID-19 vaccination, its side effects, benefits, and future implications”. Similarly, among those who were unwilling to get vaccinated in the future, we observed the highest preference for the same strategy. The lowest proportion of agreement among the non-willing group (43.9%) was to the strategy “I am willing to get vaccinated if my spiritual advisor recommended me to get the COVID-19 vaccine” (Table 4). Receiving recommendations from family and colleagues/coworkers was also not among the most preferred strategies with less than 50% agreeing or strongly agreeing to these strategies.

**Table 4:** Preference of vaccine uptake strategies by willingness to get vaccinated in the future among non-vaccinated, expressed as*N* (%)

Vaccine uptake strategies	Willingness to vaccinate in the future		
	Willing (*n* = 147)	Non-willing (*n* = 78)	*p*-value
I need to learn more about COVID-19 vaccination, its side effects, benefits, and future implications	76 [81.7]	30 [58.8]	< 0.0001
I am willing to get vaccinated if my healthcare provider recommends me to get the COVID-19 vaccine	69 [70.4]	34 [57.6]	0.167
The CDC/local health department recommended us to get the COVID-19 vaccine	74 [75.5]	30 [55.6]	< 0.042
I am willing to get vaccinated if my nurse recommended me to get the COVID-19 vaccine	74 [74.7]	31 [51.7]	0.004
I am willing to get vaccinated if my family members recommended me to get the COVID-19 vaccine	72 [75.8]	29 [45.3]	0.008
I am willing to get vaccinated if my colleagues/co-workers recommended me to get the COVID-19 vaccine	63 [67.0]	24 [45.3]	0.028
I am willing to get vaccinated if my spiritual advisor (such as pastor/priest/rabbi/imam) recommended me to get the COVID-19 vaccine	68 [72.3]	25 [43.9]	0.008

## Discussion

We used survey data to examine the COVID-19 vaccine hesitancy among vaccinated and non-vaccinated AAs living in South Georgia. We found young adults (ages 18–34) account for a disproportionate share of non-vaccinated individuals. Additionally, individuals who reported low income (household income of $25,000 or less) were more likely to be non-vaccinated. We also identified clinical barriers and myths to be the most common reasons for vaccine hesitancy among the non-vaccinated group when compared to those who were vaccinated (*p* < 0.001). Regardless of vaccination status, a majority of participants agreed that they needed to learn more about the COVID-19 vaccine. Comparatively, a smaller proportion of participants reported agreement with the willingness to get vaccinated if recommended by their colleagues or co-workers and religious and spiritual advisors.

The findings of this study are consistent with several recent systematic reviews that identified younger age and low income to be associated with higher levels of vaccine hesitancy [15, 37, 38]. Further, even among the adults who completed the primary series (i.e., those who completed the second dose for the 2-dose series COVID-19 vaccine) [39], only 31.0% of 18- to 49-year-olds have received the bivalent vaccine compared to 48.5% of 65 + years old adults and cite being busy or forgetful and mild side effects as major reasons [40]. The young adults are considered to be a relatively healthier group, and thus, they might not be a target of vaccine promotion efforts; however, our findings suggest a targeted approach that ensures the participation of young adults and low-income individuals is necessary for improvement in vaccine uptake. The findings suggest that strategies to encourage vaccine uptake in these groups should include providing additional information about the vaccine and its side effects and benefits. This information should come from trusted sources like healthcare providers, including nurses. Recent studies have also highlighted the need for such targeted communication of reliable and accurate information on the COVID-19 vaccine and its efficacy through healthcare professionals, who are viewed as trusted public figures to share such accurate information [41–44].

The findings of this study also suggest that additional information should directly address clinical risks as well as myths associated with the vaccine. Among the non-vaccinated, a majority expressed concerns related to blood clots, dying, getting sick, heart disease, pain, and high blood pressure as barriers to vaccination. Additionally, among those non-vaccinated, the majority reported myths associated with miscarriages and safety for pregnant women and the belief that the vaccine remains in the body for a long time. Even though our results come from 3 years post-vaccine rollout and public health efforts have been successful in eliminating barriers related to access (such as shortage), trust, and religion, we still observe similar vaccine barriers that we observed during the initial phase of the rollout. Multiple studies and systematic reviews using data from the initial phase of vaccine rollout have identified exposure to vaccine misinformation (referred to as myths in this study) as a leading cause of vaccine hesitancy [45–47] and exposure to conspiracy theories to have a positive association with vaccine hesitancy [15]. A study by Yang et. al. (2024) among AAs from the southern US found clinical concerns such as vaccine safety, ingredients of the vaccine, emergency approval, and inconsistent information to be prominent barriers for non-vaccinated individuals [18]. For the AA community specifically, concerns about being mistreated by the medical establishment and vaccine safety concerns are highlighted in the literature [15, 21]. Further, studies among other marginalized communities share similar barriers such as vaccine safety and its side effects [48, 49].

Most studies focused on vaccine hesitancy in the AA community, and effective strategies to increase vaccine uptake have reported a need for tailored messaging often delivered by the local faith community [50, 51]. A systematic review of vaccine hesitancy in general, not just related to COVID-19, also identified mobilization by religious leaders as an effective strategy for reducing vaccine hesitancy [52]. While more than half of the participants who were non-vaccinated in this study agreed that they were willing to get vaccinated if the recommendation came from a pastor or priest, a higher proportion reported a willingness if that recommendation came from a healthcare provider, CDC or local health department, or nurse. This suggests that messaging from a variety of sources may be needed to increase vaccine uptake.

These findings provide further insight into the dynamics of vaccine hesitancy within the AA population, specifically, providing insight into subgroups within this population and strategies for increasing vaccine uptake. With clinical barriers being the most common reason for vaccine hesitancy among the non-vaccinated in this study, programs that specifically address those concerns through education and health literacy programs may be most appropriate. Public health programs on vaccine hesitancy have focused on vaccine access and mistrust, and it appears those programs have been successful as a smaller proportion of participants in this study reported access, information, and trust barriers. As resources for vaccine promotion become constrained due to differing national priorities, it is essential to identify and focus on the most effective strategies. A review of trusted messengers and channels for COVID-19 vaccination among AA communities found the use of fact-based information through healthcare professionals along with compelling lived experiences on the COVID-19 vaccine using multiple channels to be effective [53]. Similarly, relative risk tool (RRT), a risk communication tool that presents absolute risk associated with COVID-19 and relative risk to other familiar situations such as driving, pregnancy, or sports, was found to be effective in changing risk perception and improving vaccination intent through accurate information about the risk of adverse outcome [54]. Thus, communication strategies focused on AA non-vaccinated individuals could be developed using such risk communication tools in coordination with community leaders and local healthcare providers to implement awareness campaigns. Such campaigns could provide both fact-based information on vaccine efficiency and lived/successful experiences from vaccinated and non-vaccinated individuals to empower the community to make informed decisions on vaccination intent. Similarly, organizations offering vaccines could train their healthcare workers to ask about patient concerns with receiving a vaccine, acknowledge the patients’ concerns without judgment, and provide accurate information. Additionally, a toolkit could be created with ready-made tools for community leaders that provide information on how to navigate these conversations and has reproducible materials that could be posted in faith-based organizations or other community organizations that acknowledge concerns about vaccines, provide accurate information, and demonstrate lived experiences from vaccinated people.

The study has a few limitations. Due to the cross-sectional design, we cannot establish the causality and temporality in perceptions of barriers and vaccination decisions. However, the observed differences in the perception among vaccinated and non-vaccinated participants provide support for the idea that vaccine barriers influenced the decision to receive a vaccine. Similarly, we used self-reported responses, which could be subject to measurement bias and social desirability. Despite these limitations, this study has its strengths. There are only a few studies identifying barriers to remaining non-vaccinated and strategies to vaccination even after 3 years of vaccine availability. This study incorporates the AAs living in South Georgia and explores their vaccine hesitancy perception and preference for the uptake strategies after 3 years of vaccine implementation. The study area for this research is representative of many neighborhoods around the US, where the majority of the population is AAs and non-vaccinated against COVID-19. Future research could target a large sample to allow studying sociodemographic attributes associated with perceptions of barriers and strategies and longitudinal design following the non-vaccinated group over time to further understand factors that influence the decision to vaccinate for COVID-19 as well as other annual vaccinations such as influenza in real-time. Additionally, this study can inform messaging designed to address perceived clinical barriers and myths for the COVID-19 vaccine as well as other vaccinations with higher vaccine hesitancy such as the human papillomavirus vaccination [55].

## Conclusion

Clinical concerns and myth-related barriers were the most common reasons for vaccine hesitancy, which could potentially be addressed by providing additional information on COVID-19 vaccination. These findings provide further insight into the dynamics of vaccine hesitancy within the AA community in the southern US. Many public health programs have focused on vaccine access and mistrust, which do not appear to be the leading cause of vaccine hesitancy for this population. With limited resources available, programs that specifically target such barriers through health literacy programs may be most appropriate.

## Supplementary Information

Below is the link to the electronic supplementary material.

ESM 1 (DOCX 689 KB)

## Data Availability

The data underlying this article cannot be shared publicly to maintain the privacy of individuals who participated in the study. The data will be shared on a reasonable request to the corresponding author.

## References

[1] Deslatte M. Louisiana data: virus hits blacks, people with hypertension. 2020. https://apnews.com/article/cde897293f88f788d3f3bfdb3d46c466. Accessed February 24 2023.

[2] Tiwari BB, Zhang DS. Differences in mental health status among Asian Americans during the COVID-19 pandemic: findings from the health, ethnicity, and pandemic study. Health Equity. 2022;6(1):448–53. 10.1089/heq.2022.0029. 35801151 10.1089/heq.2022.0029 PMC9257548

[3] CDC. Risk for COVID-19 infection, hospitalization, and death by race/ethnicity. 2023. https://archive.cdc.gov/www_cdc_gov/coronavirus/2019-ncov/covid-data/investigations-discovery/hospitalization-death-by-race-ethnicity.html. Accessed January 24 2024.

[4] Rossen LM, Gold JAW, Ahmad FB, Sutton PD, Branum AM. Trends in the distribution of COVID-19 deaths by age and race/ethnicity — United States, April 4–December 26, 2020. Ann Epidemiol. 2021;62:66–8. 10.1016/j.annepidem.2021.06.003. 34161794 10.1016/j.annepidem.2021.06.003 PMC8214913

[5] Tiu A, Susswein Z, Merritt A, Bansal S. Characterizing the spatiotemporal heterogeneity of the COVID-19 vaccination landscape. Am J Epidemiol. 2022;191(10):1792–802. 10.1093/aje/kwac080. 35475891 10.1093/aje/kwac080 PMC9129108

[6] Ndugga N, Hill L, Artiga S, Haldar S. Latest data on COVID-19 vaccinations by race/ethnicity. 2022. https://www.kff.org/coronavirus-covid-19/issue-brief/latest-data-on-covid-19-vaccinations-by-race-ethnicity/. Accessed September 18 2024.

[7] Kriss JL, Hung MC, Srivastav A, Black CL, Lindley MC, Lee JT, et al. COVID-19 vaccination coverage, by race and ethnicity - National Immunization Survey adult COVID module, United States, December 2020-November 2021. MMWR Morb Mortal Wkly Rep. 2022;71(23):757–63. 10.15585/mmwr.mm7123a2. 35679179 10.15585/mmwr.mm7123a2 PMC9181054

[8] Hill L, Artiga S, Ndugga N. COVID-19 cases, deaths, and vaccinations by race/ethnicity as of winter 2022. 2023. https://www.kff.org/coronavirus-covid-19/issue-brief/covid-19-cases-deaths-and-vaccinations-by-race-ethnicity-as-of-winter-2022/. Accessed January 24 2024.

[9] Aleem A, Akbar Samad AB, Vaqar S. Emerging variants of SARS-CoV-2 and novel therapeutics against coronavirus (COVID-19). StatPearls. Treasure Island (FL): StatPearls Publishing Copyright © 2024, StatPearls Publishing LLC.; 2024. https://www.ncbi.nlm.nih.gov/books/NBK570580/34033342

[10] Iqbal M, Zahidie A. Diffusion of innovations: a guiding framework for public health. Scandinavian Journal of Public Health. 2021;50(5):533–7. 10.1177/14034948211014104. 34058897 10.1177/14034948211014104

[11] D’Souza GC, Pinto CN, Exten CL, Yingst JM, Foulds J, Anderson J, et al. Understanding factors associated with COVID-19 vaccination among health care workers using the Diffusion of Innovation Theory. Am J Infect Control. 2024. 10.1016/j.ajic.2023.11.019. 38218328 10.1016/j.ajic.2023.11.019 PMC12204223

[12] Hunter-Mullis K, Macy JT, Greene A, Simon K. Perceived COVID-19 vaccine attributes associated with early adoption among adults in rural Indiana. Health Educ Res. 2022;37(6):466–75. 10.1093/her/cyac029. 36242555 10.1093/her/cyac029 PMC9619772

[13] Mo PK, Luo S, Wang S, Zhao J, Zhang G, Li L, et al. Intention to receive the COVID-19 vaccination in China: application of the diffusion of innovations theory and the moderating role of openness to experience. Vaccines (Basel). 2021;9(2). 10.3390/vaccines9020129. 10.3390/vaccines9020129 PMC7915878 33562894

[14] Rogers EM, Singhal A, Quinlan MM. Diffusion of Innovations 1. In tacks D, Salwen M, Eichhorn K, editors. An Integrated Approach to Communication Theory and Research. 3rd ed. Routledge; 2019. pp. 415–434. 10.4324/9780203710753-35

[15] Khubchandani J, Macias Y. COVID-19 vaccination hesitancy in Hispanics and African-Americans: a review and recommendations for practice. Brain Behav Immun Health. 2021;15. 10.1016/j.bbih.2021.100277. 34036287 10.1016/j.bbih.2021.100277 PMC8137342

[16] Njoku A, Joseph M, Felix R. Changing the narrative: structural barriers and racial and ethnic inequities in COVID-19 vaccination. International Journal of Environmental Research and Public Health. 2021;18(18). 10.3390/ijerph18189904. 10.3390/ijerph18189904 PMC8470519 34574827

[17] Roat C, Webber-Ritchey KJ, Spurlark RS, Lee Y-M. Black Americans receiving the COVID-19 vaccine and effective strategies to overcome barriers: an integrative literature review. J Racial Ethn Health Disparities. 2023;10(5):2577–87. 10.1007/s40615-022-01437-w. 36469286 10.1007/s40615-022-01437-w PMC9734369

[18] Yang H, Poudel N, Simpson S, Chou C, Ngorsuraches S. Important barriers to COVID-19 vaccination among African Americans in Black Belt region. J Racial Ethn Health Disparities. 2023. 10.1007/s40615-023-01583-9. 37071332 10.1007/s40615-023-01583-9 PMC10112325

[19] Adu P, Popoola T, Medvedev ON, Collings S, Mbinta J, Aspin C, et al. Implications for COVID-19 vaccine uptake: a systematic review. J Infect Public Health. 2023;16(3):441–66. 10.1016/j.jiph.2023.01.020. 36738689 10.1016/j.jiph.2023.01.020 PMC9884645

[20] Aw J, Seng JJ, Seah SS, Low LL. COVID-19 vaccine hesitancy—a scoping review of literature in high-income countries. Vaccines. 2021;9(8). 10.3390/vaccines9080900. 10.3390/vaccines9080900 PMC8402587 34452026

[21] Adeagbo M, Olukotun M, Musa S, Alaazi D, Allen U, Renzaho AMN, et al. Improving COVID-19 vaccine uptake among black populations: a systematic review of strategies. International Journal of Environmental Research and Public Health. 2022;19(19). 10.3390/ijerph191911971. 10.3390/ijerph191911971 PMC9565689 36231270

[22] CDC. Trends in United States COVID-19 deaths, emergency department (ED) visits, and test positivity by geographic area. 2024. https://covid.cdc.gov/covid-data-tracker/#trends_totaldeaths_select_00. Accessed June 20 2024.

[23] CDC. SARS-CoV-2 variant classifications and definitions. 2023. https://www.cdc.gov/coronavirus/2019-ncov/variants/variant-classifications.html. Accessed June 20 2024.

[24] United States Census Bureau. Quick facts Albany City, Georgia. 2023. https://www.census.gov/quickfacts/fact/table/albanycitygeorgia/PST045223. Accessed February 6 2024.

[25] Barry E. Days after a funeral in a Georgia town, coronavirus ‘hit like a bomb’. 2020. https://www.nytimes.com/2020/03/30/us/coronavirus-funeral-albany-georgia.html. Accessed February 6 2024.

[26] CDC. COVID-19 vaccinations in the United States. 2023. https://covid.cdc.gov/covid-data-tracker/#vaccinations_vacc-people-onedose-pop-total. Accessed February 6 2024.

[27] Georgia Department of Public Health. GA DPH vaccine distribution dashboard. 2024. https://experience.arcgis.com/experience/3d8eea39f5c1443db1743a4cb8948a9c. Accessed February 6 2024.

[28] Burke PF, Masters D, Massey G. Enablers and barriers to COVID-19 vaccine uptake: an international study of perceptions and intentions. Vaccine. 2021;39(36):5116–28. 10.1016/j.vaccine.2021.07.056. 34340856 10.1016/j.vaccine.2021.07.056 PMC8299222

[29] Crawshaw J, Konnyu K, Castillo G, van Allen Z, Grimshaw JM, Presseau J. Behavioural determinants of COVID-19 vaccination acceptance among healthcare workers: a rapid review. Public Health. 2022;210:123–33. 10.1016/j.puhe.2022.06.003. 35985082 10.1016/j.puhe.2022.06.003 PMC9192793

[30] Guidry JPD, Laestadius LI, Vraga EK, Miller CA, Perrin PB, Burton CW, et al. Willingness to get the COVID-19 vaccine with and without emergency use authorization. Am J Infect Control. 2021;49(2):137–42. 10.1016/j.ajic.2020.11.018. 33227323 10.1016/j.ajic.2020.11.018 PMC7677682

[31] Kanozia R, Arya R. “Fake news”, religion, and COVID-19 vaccine hesitancy in India, Pakistan, and Bangladesh. Media Asia. 2021;48(4):313–21. 10.1080/01296612.2021.1921963.

[32] Olagoke AA, Olagoke OO, Hughes AM. Intention to vaccinate against the novel 2019 coronavirus disease: the role of health locus of control and religiosity. J Relig Health. 2021;60(1):65–80. 10.1007/s10943-020-01090-9. 33125543 10.1007/s10943-020-01090-9 PMC7596314

[33] Coe AB, Gatewood SB, Moczygemba LR, Goode JV, Beckner JO. The use of the health belief model to assess predictors of intent to receive the novel (2009) H1N1 influenza vaccine. Innov Pharm. 2012;3(2):1–11. 10.24926/iip.v3i2.257. 22844651 10.24926/iip.v3i2.257 PMC3405550

[34] Ditsungnoen D, Greenbaum A, Praphasiri P, Dawood FS, Thompson MG, Yoocharoen P, et al. Knowledge, attitudes and beliefs related to seasonal influenza vaccine among pregnant women in Thailand. Vaccine. 2016;34(18):2141–6. 10.1016/j.vaccine.2016.01.056. 26854910 10.1016/j.vaccine.2016.01.056 PMC4811693

[35] Coman IA, Xu S, Yamamoto M. COVID-19 vaccine hesitancy: disadvantaged groups’ experience with perceived barriers, cues to action, and attitudes. Am J Health Promot. 2022;37(4):488–98. 10.1177/08901171221136113. 36306535 10.1177/08901171221136113 PMC9618917

[36] Kankaraš M, Capecchi S. Neither agree nor disagree: use and misuse of the neutral response category in Likert-type scales. METRON. 2024. 10.1007/s40300-024-00276-5.

[37] Yasmin F, Najeeb H, Moeed A, Naeem U, Asghar MS, Chughtai NU, Yousaf Z, Seboka BT, Ullah I, Lin CY, Pakpour AH. COVID-19 vaccine hesitancy in the United States: a systematic review. Front in Public Health. 2021;9:770985. 10.3389/fpubh.2021.770985. 10.3389/fpubh.2021.770985 PMC8650625 34888288

[38] Nguyen KH, Anneser E, Toppo A, Allen JD, Scott Parott J, Corlin L. Disparities in national and state estimates of COVID-19 vaccination receipt and intent to vaccinate by race/ethnicity, income, and age group among adults ≥ 18 years. United States Vaccine. 2022;40(1):107–13. 10.1016/j.vaccine.2021.11.040. 34852946 10.1016/j.vaccine.2021.11.040 PMC8598948

[39] Fast HE, Zell E, Murthy BP, Murthy N, Meng L, Scharf LG, et al. Booster and additional primary dose COVID-19 vaccinations among adults aged ≥65 years - United States, August 13, 2021-November 19, 2021. MMWR Morb Mortal Wkly Rep. 2021;70(50):1735–9. 10.15585/mmwr.mm7050e2. 34914672 10.15585/mmwr.mm7050e2 PMC8675661

[40] CDC. Concerns about bivalent COVID-19 vaccine and reasons for non-vaccination among adults who completed a primary series – omnibus survey, March 10–April 30, 2023 & household pulse survey, March 1–April 10, 2023. 2023. https://www.cdc.gov/vaccines/imz-managers/coverage/covidvaxview/pubs-resources/covid-vaccine-reasons-non-vaccination.html. Accessed April 9 2024.

[41] Begum T, Efstathiou N, Bailey C, Guo P. Cultural and social attitudes towards COVID-19 vaccination and factors associated with vaccine acceptance in adults across the globe: a systematic review. Vaccine. 2024;42(22). 10.1016/j.vaccine.2024.05.041. 38806355 10.1016/j.vaccine.2024.05.041

[42] Lee YM, Simonovich SD, Li S, Amer L, Wagner L, Hill J, et al. Motivators and barriers to COVID-19 vaccination in young adults living in the USA. Clin Nurs Res. 2023;32(6):971–82. 10.1177/10547738231177331. 37264835 10.1177/10547738231177331 PMC10261948

[43] Riggle-van Schagen C, Andrade EL, Chandarana S, Lu N, González A, Favetto C, et al. Assessing changes in COVID-19 vaccine uptake and intentions among the Brigada Digital Latino social media audience: a repeated cross-sectional study. BMC Digital Health. 2024;2(1):72. 10.1186/s44247-024-00131-7.

[44] Somefun OD, Casale M, Ronnie GH, Sumankuuro J, Akintola O, Desmond C, et al. Amplifying youth voices: young people’s recommendations for policy and practice to enhance vaccine acceptability. BMC Health Serv Res. 2024;24(1):1425. 10.1186/s12913-024-11630-8. 39558407 10.1186/s12913-024-11630-8 PMC11571648

[45] Kricorian K, Civen R, Equils O. COVID-19 vaccine hesitancy: misinformation and perceptions of vaccine safety. Hum Vaccin Immunother. 2022;18(1):1950504. 10.1080/21645515.2021.1950504. 34325612 10.1080/21645515.2021.1950504 PMC8920251

[46] Zhao S, Hu S, Zhou X, Song S, Wang Q, Zheng H, et al. The prevalence, features, influencing factors, and solutions for COVID-19 vaccine misinformation: systematic review. JMIR Public Health Surveill. 2023;9. 10.2196/40201. 36469911 10.2196/40201 PMC9838721

[47] Zimmerman T, Shiroma K, Fleischmann KR, Xie B, Jia C, Verma N, et al. Misinformation and COVID-19 vaccine hesitancy. Vaccine. 2023;41(1):136–44. 10.1016/j.vaccine.2022.11.014. 36411132 10.1016/j.vaccine.2022.11.014 PMC9659512

[48] Newman PA, Dinh DA, Nyoni T, Allan K, Fantus S, Williams CC, et al. Covid-19 vaccine hesitancy and under-vaccination among marginalized populations in the United States and Canada: a scoping review. J Racial Ethn Health Disparities. 2025;12(1):413–34. 10.1007/s40615-023-01882-1. 38117443 10.1007/s40615-023-01882-1 PMC11746967

[49] Rossi MM, Parisi MA, Cartmell KB, McFall D. Understanding COVID-19 vaccine hesitancy in the Hispanic adult population of South Carolina: a complex mixed-method design evaluation study. BMC Public Health. 2023;23(1):2359. 10.1186/s12889-023-16771-9. 38017470 10.1186/s12889-023-16771-9 PMC10685550

[50] Abdul-Mutakabbir JC, Casey S, Jews V, King A, Simmons K, Hogue MD, et al. A three-tiered approach to address barriers to COVID-19 vaccine delivery in the Black community. Lancet Glob Health. 2021;9(6):e749–50. 10.1016/S2214-109X(21)00099-1. 33713634 10.1016/S2214-109X(21)00099-1 PMC7946412

[51] Privor-Dumm L, King T. Community-based strategies to engage pastors can help address vaccine hesitancy and health disparities in Black communities. J Health Commun. 2020;25(10):827–30. 10.1080/10810730.2021.1873463. 33719889 10.1080/10810730.2021.1873463

[52] Jarrett C, Wilson R, O’Leary M, Eckersberger E, Larson HJ. Strategies for addressing vaccine hesitancy – a systematic review. Vaccine. 2015;33(34):4180–90. 10.1016/j.vaccine.2015.04.040. 25896377 10.1016/j.vaccine.2015.04.040

[53] Rabin Y, Kohler RE. COVID-19 vaccination messengers, communication channels, and messages trusted among black communities in the USA: a review. J Racial Ethn Health Disparities. 2025;12(1):134–47. 10.1007/s40615-023-01858-1. 37947953 10.1007/s40615-023-01858-1 PMC11345940

[54] Byerley CON, Horne D, Gong M, Musgrave S, Valaas LA, Rickard B, et al. An effective COVID-19 vaccine hesitancy intervention focused on the relative risks of vaccination and infection. Sci Rep. 2024;14(1):7419. 10.1038/s41598-024-57841-1. 38548828 10.1038/s41598-024-57841-1 PMC10978892

[55] White JL, Grabowski MK, Rositch AF, Gravitt PE, Quinn TC, Tobian AAR, et al. Trends in adolescent human papillomavirus vaccination and parental hesitancy in the United States. J Infect Dis. 2023;228(5):615–26. 10.1093/infdis/jiad055. 36869689 10.1093/infdis/jiad055 PMC10469123

