# Supplementary material for: Barriers to COVID-19 Vaccination and Perceptions Around Vaccination Uptake Strategies Among African Americans Living in the US South: Opportunities for Public Health Program Intervention
Source: J Racial Ethn Health Disparities. 2025 Apr 28;13(3):2476–86. doi: 10.1007/s40615-025-02433-6 (PMC13157365; doi:10.1007/s40615-025-02433-6)
Supplement: Supplementary file 1 — (DOCX 689 KB) [file 40615_2025_2433_MOESM1_ESM.docx]

# **APPENDIX**

## **Appendix 1**

Appendix Table 1: Survey Based Research on COVID-19 Vaccine Hesitancy among African Americans in the US

| Title | Authors (Year) | Research Objective | Study population | Population addressed/Geographic region | Article DOI |
| --- | --- | --- | --- | --- | --- |
| Important Barriers to COVID-19 Vaccination Among African Americans in Black Belt Region | Yang et al. (2023) | Identify important barriers to COVID-19 vaccination among African Americans living in the Black Belt region | 808 African Americans | Black Belt Region | <https://doi.org/10.1007/s40615-023-01583-9> |
| Current Health Care Experiences, Medical Trust, and COVID-19 Vaccination Intention and Uptake in Black and White Americans | Martin K.J. et al. (2022) | Assess the relationship between healthcare experiences, historical knowledge of medical mistreatment, medical trust, and COVID-19 vaccination intention and uptake in Black and White Americans | Black and White Americans: 297 in Study 1 and 12,757 in Study 2 | USA | <https://doi.org/10.1037/hea0001240> |
| HIV Care Engagement Is Not Associated with COVID-19 Vaccination Hesitancy during the Initial Peak of the COVID-19 Pandemic among Black Cisgender Sexual Minority Men and Transgender Women in the N2 COVID Study | Duncan D.T. et al. (2023) | Examine whether an association exists between HIV care engagement (i.e., current PrEP or ART use) and COVID-19 vaccination hesitancy among Black cisgender sexual minority men and transgender women at the initial peak of the pandemic | 222 Black Cisgender Sexual Minority Men and Transgender Women | Chicago, USA | <https://doi.org/10.3390/vaccines11040787> |
| Assessing the Impact of COVID-19 Phased Vaccine Eligibility on COVID-19 Vaccine Intent among African Americans in Southeastern Louisiana: A Community-Based, Cohort Study | Al-Dahir S. et al. (2022) | Determine the impact of vaccine eligibility on the likelihood of vaccine uptake among African Americans in southeastern Louisiana | 487 African Americans | Southeastern Louisiana, USA | <https://doi.org/10.3390/ijerph192416737> |
| The Roles of Social Media Use and Medical Mistrust in Black Americans' COVID-19 Vaccine Hesitancy: The RISP Model Perspective | Nah S. et al. (2023) | Explore the roles of medical mistrust and social media as a source of risk information in Black Americans’ vaccine hesitancy | 1136 African Americans | USA | <https://doi.org/10.1080/10410236.2023.2244169> |
| Moral Foundations Predict COVID-19 Vaccine Hesitancy: Evidence from a National Survey of Black Americans | Nan X. et al. (2022) | Examine the role of moral values in predicting COVID-19 vaccine hesitancy among Black Americans | 1497 Black Americans | USA | <https://doi.org/10.1080/10810730.2022.2160526> |
| COVID-19 Morbidity, Vaccine Side Effects, and Vaccine Hesitancy among African Americans | Zhang F. and Marvel J.D. (2022) | Investigate vaccination intentions in Black communities and explore behavioral mechanisms involved in vaccination decisions | 547 African Americans | USA | <https://doi.org/10.1061/(asce)nh.1527-6996.0000580> |
| Correlates of COVID-19 Vaccine Uptake in Black Adults Residing in Allegheny County, PA | Hill A.V. et al. (2023) | Investigate factors related to COVID-19 vaccine uptake and access among black residents in Allegheny County, PA | 397 Black Adults | Allegheny County, PA, USA | <https://doi.org/10.1089/heq.2022.0215> |
| Barriers to COVID-19 Vaccination and Perceptions Around Vaccination Uptake Strategies Among African Americans Living in the US South: Opportunities for Public Health Program Intervention | Our Paper | Identify COVID-19 vaccination barriers from the non-vaccinated group three years post-vaccine availability and the preferred vaccine promotion strategies by non-vaccinated individuals to break the vaccine barriers | 1471 African Americans | Albany, Georgia |  |

## **Appendix 2**

Total Surveyed Participants between October 2022 and July 2023,

**N = 2058**

African American (AA) Participants,

**N = 1908**

Missing Race/Ethnicity information,

**n = 39**

Participants identifying with race/ethnicity other than AA,

**n = 111**

Complete Information on Vaccine Barriers and Uptake Strategies,

**N = 1715**

Missing Information on Vaccine Barriers and Uptake Strategies,

**n = 193**

Complete Information on Covariates,

**N = 1620**

Missing/Incorrect Information on Covariates, **n = 95**

Final Sample,

**N = 1471**

Missing Information on Self-reported Vaccination Status, **n = 149**

**Appendix**

**Appendix Figure 2: Sample Selection Procedure for the Study**

## **Appendix 3**

##
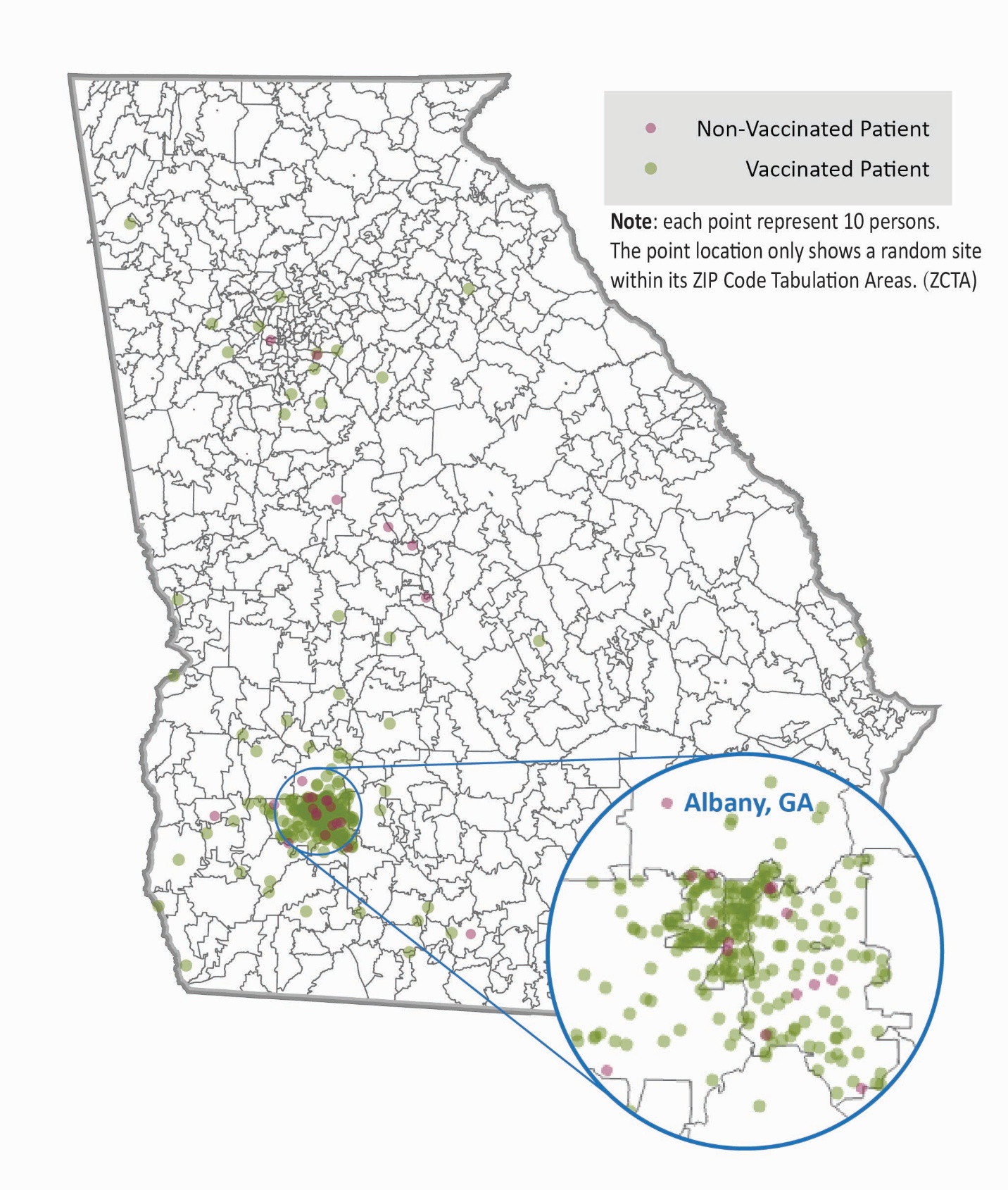


## **Appendix Figure 3: Distribution of Study Participants by their Vaccination Status**

## **Appendix 4**

## **List of Vaccine Barriers Used in the Study**

| **Vaccine Barriers** | **Measures** |
| --- | --- |
| **Clinical Barrier** | Blood clots from the COVID-19 vaccine are of concern |
|  | Dying from the COVID-19 vaccine is of concern |
|  | Getting sick from multiple doses of COVID-19 vaccine is of concern |
|  | Pain from/related to the COVID-19 vaccine is of concern. |
|  | Heart disease from the COVID-19 vaccine is of concern |
|  | High blood pressure from the COVID-19 vaccine is of concern |
|  |  |
| **Myths** | The COVID-19 vaccine leads to miscarriages and is not appropriate for pregnant women |
|  | COVID-19 vaccine components remain in the human body for a long period |
|  | COVID-19 vaccine implants a “computer chip” in the body |
|  | COVID-19 vaccine is NOT an effective way to protect against COVID-19. |
|  |  |
| **Access Barrier** | It is inconvenient to get the COVID-19 vaccine |
|  | There is a shortage of the COVID-19 vaccine |
|  | The clinics/venues that provide the COVID-19 vaccine are too far away |
|  | I don’t know how to get ahold of the COVID-19 vaccine |
|  |  |
| **Information Barrier** | I don’t have enough information to decide whether to take COVID-19 vaccine or no |
|  | I am confused by the information about the COVID-19 vaccines |
|  | I don’t know where I can get accurate information about the COVID-19 vaccines |
|  | I don't know where I can get trustworthy information about the COVID-19 vaccines |
|  |  |
| **Trust Barrier** | I don’t trust vaccines in general |
|  | I don’t trust the government agencies that approved the COVID-19 vaccines |
|  | I don’t trust media that recommend the COVID-19 vaccines |
|  | I don’t trust the pharmaceutical companies that manufacture the COVID-19 vaccines |
|  | I don’t trust the scientists/professionals who recommend the COVID-19 vaccines |
|  |  |
| **Religious Barrier** | As long as I am faithful to my God and/or my religion, I am protected from COVID-19, therefore I do not need the COVID-19 vaccine |
|  | It is better to use natural preventive methods (e.g., essential oils, other natural drinks, tonics, etc.) to prevent getting infected with COVID-19 than getting the vaccine |
|  | It is better to use spiritual/holy preventive measures (e.g., holy water, holy oil, cross, holy amulets, etc.) to prevent getting sick from COVID-19 than to get the COVID-19 vaccine |
|  | It is better to get sick of COVID-19 and for your body to fight it off, building natural immunity than getting the vaccine |
|  | The COVID-19 vaccines ingredients are banned by my religion, therefore, I cannot get the COVID-19 vaccine |

**Appendix 5**

**List of Vaccine Promotion Strategies**

1. I need to learn more about COVID-19 vaccination, its side effects, benefits, and future implications
2. I am willing to get vaccinated if My healthcare provider recommends me to get the COVID-19 vaccine
3. The CDC/local health department recommended us to get the COVID-19 vaccine
4. I am willing to get vaccinated if My nurse recommended me to get the COVID-19 vaccine
5. I am willing to get vaccinated if My family members recommended me to get the COVID-19 vaccine
6. I am willing to get vaccinated if My colleagues/co-workers recommended me to get the COVID-19 vaccine
7. I am willing to get vaccinated if My spiritual advisor (such as pastor/priest/rabbi/imam) recommended me to get the COVID-19 vaccine

## **Appendix 6**

## **Appendix Table 6: Proportion of Non-Vaccinated Participants with Agreement Perception on Vaccine Uptake Strategies by Demographic Characteristics, expressed as N [%]**

|  | **Vaccine Uptake Strategies** | | | | | | |
| --- | --- | --- | --- | --- | --- | --- | --- |
| **Demographic Characteristics** | **The CDC/local health department recommended us to get the COVID-19 vaccine** | **I am willing to get vaccinated if My healthcare provider recommends me to get the COVID-19 vaccine** | **I am willing to get vaccinated if My nurse recommended me to get the COVID-19 vaccine** | **I am willing to get vaccinated if My spiritual advisor (such as pastor/priest/rabbi/imam) recommended me to get the COVID-19 vaccine** | **I am willing to get vaccinated if My family members recommended me to get the COVID-19 vaccine** | **I am willing to get vaccinated if My colleagues/co-workers recommended me to get the COVID-19 vaccine** | **I need to learn more about COVID-19 vaccination, its side effects, benefits, and future implications** |
| **Gender** |  |  |  |  |  |  |  |
| Male | 34 [68.0] | 40 [69.0] | 39 [68.4] | 32 [64.0] | 37 [72.5] | 31 [60.8] | 35 [74.5] |
| Female | 74 [69.2] | 67 [64.4] | 69 [63.9] | 63 [59.4] | 68 [64.2] | 60 [58.3] | 75 [72.8] |
| **Age Groups** |  |  |  |  |  |  |  |
| 18-24 | 31 [75.6] | 27 [69.2] | 28 [68.3] | 25 [67.6] | 28 [68.3] | 27 [64.3] | 33 [82.5] |
| 25-34 | 21 [56.8] | 22 [57.8] | 24 [61.5] | 19 [50.0] | 25 [67.6] | 23 [63.9] | 24 [68.6] |
| 35-64 | 25 [71.4] | 22 [61.1] | 24 [61.5] | 20 [55.6] | 22 [62.9] | 21 [58.3] | 24 [70.6] |
| 65+ | 18 [78.3] | 19 [79.2] | 17 [73.9] | 17 [77.3] | 18 [75.0] | 12 [60.0] | 18 [78.3] |
| **Education** |  |  |  |  |  |  |  |
| Less than High School | 23 [85.2] | 24 [82.8] | 24 [82.8] | 23 [82.1] | 24 [85.7] | 20 [71.4] | 26 [89.7] |
| High School | 68 [64.8] | 65 [60.2] | 67 [60.4] | 56 [54.4] | 66 [62.3] | 56 [55.4] | 68 [68.7] |
| Bachelor’s degree or higher | 5 [71.4] | 5 [71.4] | 4 [57.1] | 5 [71.4] | 5 [83.3] | 4 [57.1] | 7 [87.5] |
| **Income** |  |  |  |  |  |  |  |
| Less than 25,000$ | 69 [75.8] | 64 [71.9] | 67 [72.8] | 59 [67.8] | 67 [73.6] | 56 [62.9] | 74 [81.3] |
| 25,000$-49,900$ | 30 [55.6] | 33 [55.9] | 32 [53.3] | 27 [49.1] | 29 [55.8] | 25 [49.0] | 30 [62.5] |
| 50,000$-99,900$ | 7 [77.8] | 8 [75.0] | 7 [70.0] | 8 [72.7] | 8 [72.7] | 9 [75.0] | 5 [55.6] |
| 100,000$ or higher | 2 [66.7] | 1 [50.0] | 2 [66.7] | 1 [33.3] | 1 [33.3] | 1 [50.0] | 1 [50.0] |
| **Children** |  |  |  |  |  |  |  |
| Yes | 50 [74.6] | 51 [70.8] | 52 [69.3] | 43 [61.4] | 45 [64.3] | 39 [56.5] | 48 [75.0] |
| No | 58 [64.4] | 56 [62.2] | 56 [62.2] | 52 [60.5] | 60 [69.0] | 52 [61.2] | 62 [72.1] |
| **Elderly** |  |  |  |  |  |  |  |
| Yes | 21 [77.8] | 22 [68.8] | 21 [67.7] | 22 [66.7] | 27 [77.1] | 22 [66.7] | 25 [80.6] |
| No | 86 [66.7] | 84 [65.1] | 86 [64.7] | 72 [59.0] | 77 [63.6] | 68 [56.7] | 84 [71.2] |
| **Chronic Condition** |  |  |  |  |  |  |  |
| No Condition | 54 [68.4] | 50 [63.3] | 56[69.1] | 49 [70.1] | 54 [70.1] | 50 [70.8] | 56 [70.9] |
| At least 1 condition | 22 [81.5] | 21 [80.8] | 20[76.6] | 21 [81.8] | 20 [76.9] | 15 [83.3] | 22 [75.9] |
